# The Mississippi Valley Medical Association

**Published:** 1887-08

**Authors:** 


### THE MISSISSIPPI VALLEY MEDICAL ASSOCIATION.

THE Thirteenth Annual meeting of this society occurred July 13th, 14th, and 15th, 1887, at Crab Orchard Springs, Kentucky.

Dr. John E. Link read a paper detailing three cases of gun-shot wounds of the abdomen; two recovered without surgical interference and one with such interference, and he claimed:

1. An abdominal wound should be probed only with a grooved-director as a guide to the scalpel.

2. Any wound of this cavity and viscera may recover with fixation, narcotization, and abstinence from food.

3. The cavity of the abdomen may be invaded by the surgeon in recent injuries as well as for other conditions. His use of opium was to relieve pain and to secure rest for the bowel.

In the discussion which followed, the weight of opinion was in favor of laparotomy in all cases of penetrating wounds of the abdomen. The majority who discussed the paper believed in the use of opium. Some condemned it, and advocated purgatives according to Tait's method,

The annual address of the president of the society, Dr. I. N. Love, under the title "The Pressing Needs of the Profes sion," discussed the necessity for a highe- standard in medical education, the exr cessive number of medical colleges in this country, and the desirability of a union of all schools of medicine upon a common and broad basis.

The paper of Dr. Preston Scott, on "The Use of Chloroform During Labor," advocated a much more general use of the drug than is common. In the discussion which followed, the proposition was strongly indorsed. Some of those who discussed the paper thought fatty heart an absolute contra indication, others did not. Some thought the use of morphia with the chloroform very desirable.

Dr. George J. Cook read a paper on "Chronic Constipation," in which he classed all such cases as atonic, or obstructive. He considers disregard of natural laws the chief cause of the atonic variety, and large injections of hot water and massage along the colon the best treatment for it.

The most frequent causes of the obstructive variety, he thinks, are irritability of the sphincter ani muscle, and contraction at the upper end of the rectum.

Those taking part in the discussion of this paper were uniformly of the opinion that dilatation of the sphincter ani is the proper treatment for the obstructive variety of the disease. The degree to which dilatation of the sphincter should be carried was not generally stated by those discussing the subject.

Dr. L. S. McMurtry presented a specimen from a case of pyosalpinx, with removal four days after confinement. The temperature had been 105° F., and at the time of the operation was 103.5° F. The patient made a good recovery. The author of the paper thought the case of special interest for the reasons: 1. The history of the case indicated that the pyosalpinx was caused by a gonorrhœa, and was of long standing. 2. That the case demonstrates that conception may occur with unilateral pyosalpinx present.

Dr. A. C. Bernays presented two pathological specimens, one, the pyloric end of the stomach and part of the contiguous duodenum, removed because it was carcinomatous. In the operation of removal the ductus communis choledocus and the pancreatic duct "were torn off," but "were carefully stitched into the line of union on the posterior surface." The patient died eighteen hours after the operation. The autopsy showed "that there had been no hæmorrhage, the stomach held water, and bile discharged into the bowel."

The second specimen was a fœtus with a tail-like projection, and homologous arches, such as are found in fishes. The case was reported because of its bearing upon the theory of evolution.

Dr. Y. H. Bond read a paper upon the general subject of removal of the ovaries, asserting that the operation is too often unnecessarily done.

D. J. B. Green read a paper on "Gonorrhœal Peritonitis," in which he claimed that the infection does travel as far as the peritoneum. He reported three such cases in which abdominal section was beneficially made. The discussion of these papers developed a variety of opinions.

Dr. G. W. McCaskey read a paper in which he described and advocated an apparatus of his own construction for intra-pulmonary medication by means of vapors at high temperatures.

Several other papers were also read and discussed.

The association adopted a revised constitution, and elected the following officers for the year 1887: Dudley S. Reynolds, President; A. Dunlap, Y. H. Bond, A. R. Jenkins, H. C. Fairbrother, D. H. Thompson, Vice-Presidents; J. Lucius Gray, Permanent Secretary; A. H. Ohmann-Dumesnil, Treasurer.

The society adjourned to meet in St. Louis in September, 1888.

